# Rapid fluorescence immunoassay of benzo[*a*]pyrene in mainstream cigarette smoke based on a dual-functional antibody–DNA conjugate

**DOI:** 10.1039/c8ra04915g

**Published:** 2018-08-21

**Authors:** Ziyan Fan, Zhonghao Li, Shanshan Liu, Fei Yang, Zhaoyang Bian, Ying Wang, Gangling Tang, Qinxiao Zhao, Huimin Deng, Shili Liu

**Affiliations:** China National Tobacco Quality Supervision and Test Center No. 2 Fengyang Street, High and New Technology Industries Development Zone Zhengzhou 450001 China hmdeng_2015@163.com liushili@sdu.edu.cn +86-371-67672625 +86-531-88382502 +86-371-67672960 +86-531-88382579; School of Basic Medical Science, Shandong University Jinan Shandong 250012 China

## Abstract

Benzo[*a*]pyrene (BaP) is considered as one of the most carcinogenic pollutants in cigarette smoke. The development of simple and sensitive BaP screening methods can help assess the risk of cigarette exposure to the human body rapidly. In this report, a rapid fluorescence immunoassay (RFIA) method for the detection of BaP is proposed, the core of which is the synthesis of bifunctional covalent antibody–DNA conjugates for target recognition and signal amplification. Based on the optimization of the SYBR Green I and PAH–BSA concentrations, as well as DNA–antibody immune complex's dilution in the RFIA system, a serial dilution of BaP was tested with this method. The results showed that the linear working range of the RFIA for BaP is 0.46 to 333 ng mL^−1^, which is much wider than traditional ELISA. The detection limit was 0.32 ng mL^−1^, which was more sensitive than other methods such as the redox-labeled electrochemical immunoassay method and the competitive piezoelectric biosensor. Then the cross-reactions (CR) of other PAHs in cigarette smoke were evaluated using this RFIA and found that the cross-reactions of naphthalene, anthracene, and pyrene were very low (<1%). The cross-reaction in this RFIA system can be reduced by improving the specificity of the antibody. To the best of our knowledge, this is the first time that the BaP in mainstream cigarette smoke was tested; the RFIA demonstrates fast and simple experimental manipulations and better working curves and sensitivity.

## Abstract 2

Benzo[*a*]pyrene (BaP) is considered as one of the most carcinogenic pollutants in cigarette smoke.

## Graphical abstract

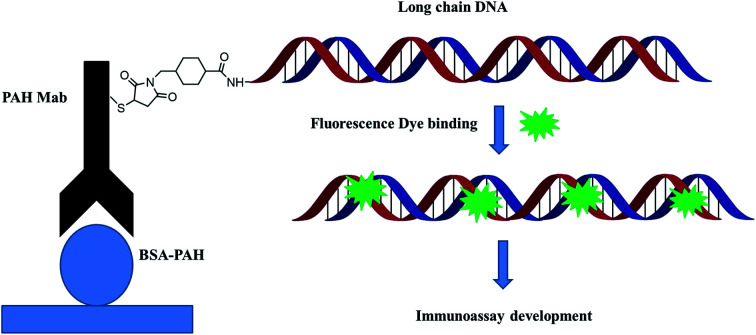

## Introduction

Smoking is considered to be the main cause of lung cancer and other related diseases. There are approximately 1.3 billion smokers worldwide, and the number of annual deaths caused by tobacco will increase from about 5 million in 2010 to more than 10 million in a few decades.^1^ Of the thousands of compounds contained in cigarette smoke, polycyclic aromatic hydrocarbons (PAHs) have attracted attention due to their toxicological effects. According to the report of the International Agency for Research on Cancer (IARC), 10 oncogenic PAHs have been identified in cigarette smoke.^2^ Among these PAHs, benzo[*a*]pyrene (BaP) is considered to be one of the most carcinogenic pollutants, and previous studies have found that the level of BaP per cigarette in Canadian cigarettes ranged from 3.36 to 28.39 ng.^3^ Toxicological studies have shown that BaP can be metabolized to the toxic compound benzo[*a*]pyrene-7,8-dihydrodiol-9,10-epoxide *via* cytochrome P450 *in vivo*, which can cause genetic damage through the formation of adducts of DNA, and thereby promotes cancer progression.^4^ In view of the toxicological effects of BaP, BaP has been listed as one of the 9 poisons that are mandatory to be reduced in cigarette smoke in a World Health Organization report. Therefore, there is a huge demand for routine screening of BaP for assessing the health risks of people exposed to cigarette smoke, as well as the effective management of toxic components of tobacco and tobacco products.

Traditionally, PAHs are analyzed by chromatographic techniques including high performance liquid chromatography (HPLC), gas chromatography mass spectrometry (GC-MS) and liquid chromatography tandem mass spectrometry (LC-MS/MS).^5–8^ Due to the low abundance of polycyclic aromatic hydrocarbons in cigarette smoke and the complex composition of cigarette smoke, these methods are always accompanied by cumbersome pre-concentration and purification pretreatment procedures, thus making these methods to a certain extent both expensive and time consuming.

In addition, targeting BaP as a method of analysis also includes the application of a series of monoclonal antibodies generated by BaP derivatives,^9–11^ and then a variety of immunochemical detection methods were developed for rapid detection of PAHs, such as enzyme-linked immunosorbent assay (ELISA), molecular imprinting biosensors, surface Plasma resonance immunosensors, fluorescent immunoassays and electrochemical immunoassays.^12–16^ Among these methods, fluorescent immunoassay has become a promising method due to the recent development of new fluorescent labeling techniques such as quantum dots, carbon nanotubes, and DNA labels, which have been used to improve the stability and sensitivity.^17–19^ Among these new fluorescence labeling techniques, fluorescence immunoassays employing antibody–DNA/dye immune complexes make fluorescence amplification possible and more compatible with protein chips and lateral flow immunoassays. This strategy is used to analyze environmental organic pollutants and the detection limit can reach pg mL^−1^ to ng mL^−1^.^20,21^ However, this signal amplification strategy requires the biotin labeling of antibodies and DNA and the bridging of streptavidin, thus complicating detection. Compared with noncovalent coupling between DNA and protein using biotin–(strept)avidin interaction, covalent conjugation with DNA allows proteins to be modified with a synthetically accessible, robust tag, to realize combined properties. Therefore, this technique has recently been widely used, such as designing and developing high-performance biosensors, as well as basic research on molecular recognition and DNA nanostructures.^22^ Stadler *et al.* synthesized a fluorescent DNA duplex covalently attached with multiple thiazole orange and antibody and used it for intracellular fluorescence imaging of centrosomes in Drosophila embryos.^23^ Seymour *et al.* also developed a novel protein microarray using DNA-binding antibodies for the rapid and sensitive detection of whole viruses.^24^ Moreover, Wold *et al.* reported a DNA encoded antibody microarray utilizing site-specific antibody–oligonucleotide covalent conjugates for capture and detection of SK-BR-3 cells (Her2+ breast cancer cells).^25^ GC/MS and fluorescence immunoassay were compared in a previous study, in which the fluorescence immunoassay methods used were enzyme immunoassay (EIA) and fluorescence polarization immunoassay (FPIA), and the substances they measured were plasma tHcy concentrations.^26^ In terms of accuracy, the results demonstrated that fluorescence immunoassay agreed excellent with GC-MS for both fasting and post-methionine load tHcy concentrations. GC-MS and FPIA are the closest methods for the detection of tHcy concentration, with 95% of FPIA values being 19% to 24% higher than the corresponding GC-MS results, indicating agreement between GC-MS and FPIA. However, the disadvantage of GC-MS is few chromatographic methods are fully automated, and in all cases, the number of samples tested per day is relatively low. In contrast, immunoassays are typically fully automated and have the potential for high daily detection.

Motivated by the strategy of covalent conjugation between DNA and antibody, in the present study, a long chain DNA was directly conjugated to the BaP monoclonal antibody by chemical covalent coupling to form a dual-functional antibody–DNA conjugate for recognition and signal amplification. The clever thing about this method is that antibodies used in immunoassays were directly or indirectly barcoded with a synthetic nucleic acid strand instead of being conjugated to a reporter enzyme such as horse radish peroxidase or alkaline phosphatase. The barcode analogy is useful when considering the obvious advantages over other methods such as redox-labeled electrochemical immunoassay, and piezoelectric biosensor: signal amplification can be achieved through long-chain DNA adsorption of dyes; and barcode analogy provides quantitative information from different biomarkers in real-time PCR.

**Scheme 1:**
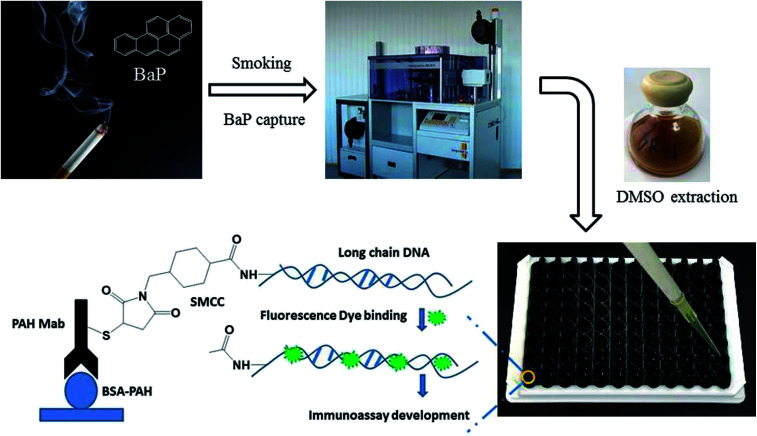


Based on this, a rapid fluorescence immunoassay (RFIA) method was developed for BaP detection in mainstream cigarette smoke (illustrated in Scheme 1). Unlike ELISA, this format provides a wider linearity and it is more compatible with the current biochip technology and lateral flow immunoassay. The proposed RFIA method can be readily developed into immunoassay for the simultaneous detection of multiple analytes in a single sample as well.

## Results and discussion

### The principle of the RFIA

**Fig. 1:**
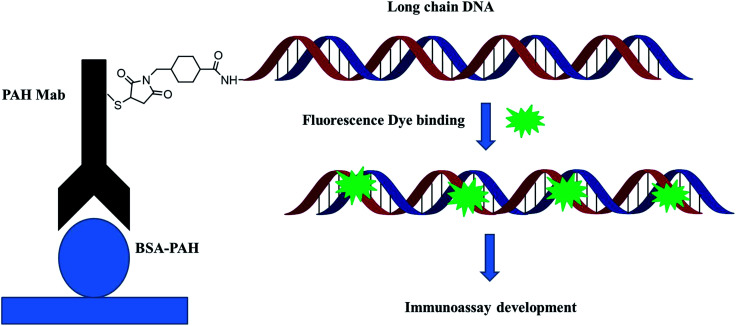
Schematic illustration of the synthetic covalent BaP antibody–DNA conjugates.

The most commonly used method of detection of PAHs is chromatographic techniques, but due to the usual by cumbersome pre-concentration and purification pretreatment procedures, making these methods to a certain extent both expensive and time consuming. As an improved detection method, our previous studies employed long-chain DNA attached to antibodies by streptavidin–biotin interaction as a carrier for fluorescence signal amplification. However, this strategy requires a two-step reaction, where the biotin-labeled antibody binds to avidin and avidin-antibody binds biotin-labeled DNA, complicating the assay and affecting detection accuracy. To simplify the immunoassay procedure, in this study, we developed a RFIA method using synthetic covalent BaP antibody–DNA conjugates for recognition and signal amplification by using the reported detection strategy of antibody–DNA complexes.^25,27,28^ As illustrated in Fig. 1, the –NH_2_-modified reporter DNA and BaP antibody were activated with sulfo-SMCC and SATA, respectively; and the two activated molecules then react to form an immune complex covalently coupled with reporter DNA and BaP antibodies for immunodetection of BaP. The immune complex contains BaP antibody for recognizing the analytes and DNA for binding the fluorescent dye, so it is a dual function unit that combines small molecule recognition and signal amplification. The length of the DNA strand was optimized in previous studies.^20^ A competitive detection strategy was adopted in this study; in which BaP was covalently immobilized on the BSA as a detection unit, the BaP to be tested was mixed with the immune complex and the dye and then incubated with the detection unit. The BaP to be measured in the solution competes with the BSA–BaP for an immune complex, thereby achieving a competitive detection with high sensitivity.

### Characterization of the long chain DNA and antibody–DNA conjugate

**Fig. 2:**
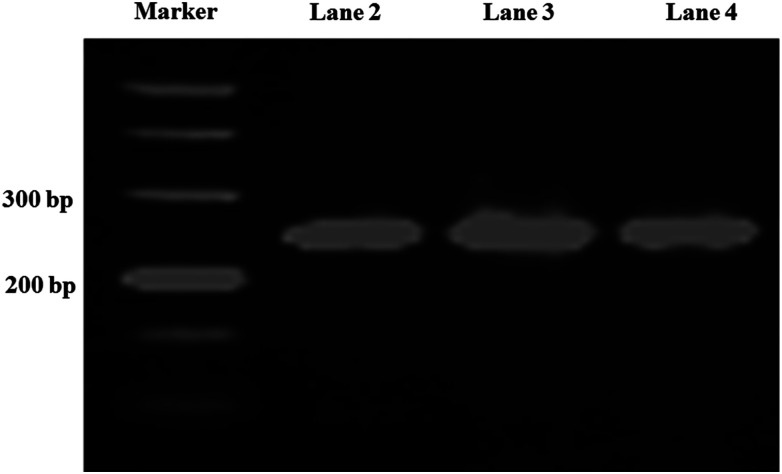
Agarose gel electrophoresis of the PCR products. Lane 1, DNA marker; lane 2, 3, 4 are PCR products.

Long-chain DNA is used as a fluorescent dye carrier, so a suitable length is critical. In traditional quantitative real-time immuno-PCR, a DNA reporter of 100–300 base pair was commonly used.^29,30^ Therefore, a 251 base-pair DNA fragment from *Phytophthora parasitica* var. *nicotianae* was synthesized for binding fluorescence dye to amplify the detection signal in this study. To achieve a controlled DNA–antibody cross-linking strategy, –NH_2_ was introduced into long-chain DNA with –NH_2_ modified primers and a PCR amplification strategy. In order to minimize the influence of the steric hindrance, an intermediate carbon chain containing 12-CH_2_– was introduced between –NH_2_ and bases. The sense primer was designed as 5′-NH_2_–C_12_-TGAACGCATATTGCACTTCC-3′. The PCR product was characterized by agarose gel electrophoresis, and the result was shown in Fig. 2. From the gel electrophoresis images, it can be seen that there was a clear single band between the 200 and 300 bp bands of the DNA ladder, and no other bands are present, indicating that the PCR product was what we expected, and the purity and concentration were high.

**Fig. 3:**
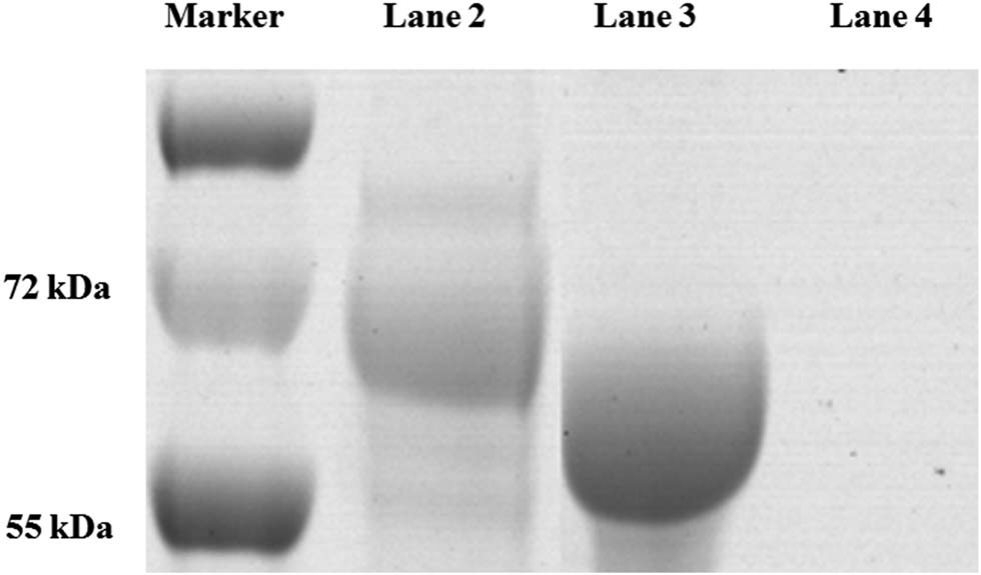
The antibody–DNA conjugate was characterized by SDS-PAGE. Lane 1, protein marker; lane 2, DNA–antibody conjugate; lane 3, DNA and antibody.

The process of covalent binding of DNA–antibodies is achieved by a reaction of the sulfhydryl (–SH) on the antibody with the –NH_2_ at the end of the DNA chain and activated by the NHS ester. This reaction is a rapid and controlled antibody coupling process. Sulfo-SMCC is a bi-functional crosslinker which contains an *N*-hydroxysuccinimide (NHS) ester and a maleimide group. The NHS ester reacts with –NH_2_ on the DNA, and the maleimide group reacts with the BaP antibody through the sulfhydryl (–SH), which is introduced by treating the antibody with SATA in advance. The DNA was conjugated to the heavy chain, total molecular weight of the complex is 221 kDa, the antibody–DNA conjugate was characterized by SDS-PAGE (Fig. 3). Since the sample was subjected to a reducing agent treatment prior to SDS-PAGE, the antibody was reduced to two fragments, the heavy chain (55 kDa) and the light chain (25 kDa).^31^ Observation of the stained SDS-PAGE pattern revealed that the band appeared in the DNA–antibody conjugate solution (lane 2) had a molecular weight of approximately 72 kDa, which was significantly greater than that in the mixture of DNA and antibody (lane 3), indicating that the BaP antibody has been covalently coupled to DNA.

### Optimization of the RFIA system

**Fig. 4:**
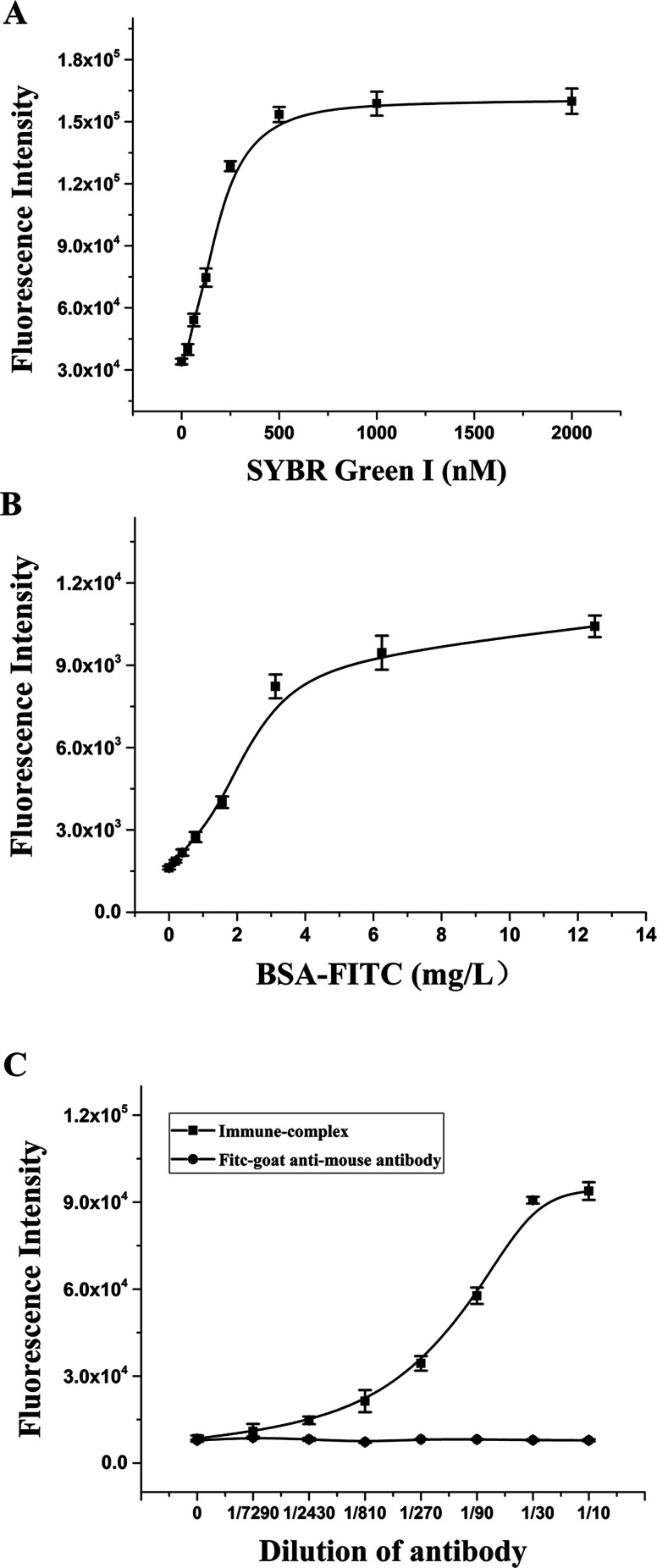
Optimization of the RFIA system. SYBR Green I (A), PAH–BSA (B) and antibody–DNA conjugate (C) concentrations were optimized.

To establish the RFIA method for immunoassay of BaP, we first investigated the ability of DNA–antibody immune complexes to bind to fluorescent dyes. The immune complexes were diluted 10-fold and titrated with SYBR Green I. As illustrated in Fig. 4A, the fluorescence increased as the SYBR Green I concentration increased from 0 to 2000 nmol L^−1^, and reached a plateau at approximately 500 nmol L^−1^, indicating that 500 nmol L^−1^ SYBR Green I can saturate the binding capacity at this immune complex concentration. The optimal concentration of PAH–BSA immobilized on the microplate was studied by FITC-labeled BSA. As shown in Fig. 4B, as the concentration of BSA-FITC increased from 0 to 12.5 μg mL^−1^, the fluorescence intensity increased and reached a plateau after 6.25 μg mL^−1^, indicating that the adsorption of BSA-FITC reached saturation. Therefore, 10 μg mL^−1^ was used as the immobilized concentration of PAH–BSA in the RFIA system to ensure the immobilization efficiency of PAH–BSA.

To determine the optimal concentration of antibody–DNA conjugate to be used for the RFIA detection, we performed a series of dilutions of antibody–DNA conjugates from 1/7290 to 1/10. The diluted immune complex solution was added to the PAH–BSA-coated plate for incubation, followed by incubation with 500 nmol L^−1^ SYBR Green I, and the fluorescence intensity of the immune complex was detected after washing. The result demonstrated that the fluorescence intensity increased significantly after the immune complex was added to the plate, and the fluorescence intensity showed a linear increase trend at a dilution of 1/300 to 1/10. As a control, no increase in fluorescence was observed with the FITC-labeled goat anti-mouse antibody, indicating that the system has excellent specificity. In order to obtain a sensitive response to free PAH by competition detection, a 1/50 dilution solution of the immune complex was used for the immunoassay.

### Detection of BaP in mainstream smoke of cigarettes with the RFIA

**Fig. 5:**
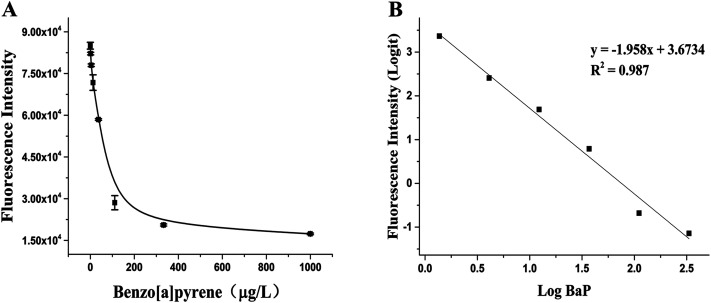
Detection of BaP in mainstream smoke of cigarettes with the RFIA. (A) Competitive curve of BaP in cigarette mainstream smoke; (B) log–logit fitting of the competitive curve.

BaP is considered to be one of the most carcinogenic PAHs, so the control of tobacco has attracted people's attention, especially in the control of the emission of harmful cigarette smoke components.^32^ Under the optimal experimental conditions determined after optimization, the RFIA was used to analyze BaP in cigarette mainstream smoke. The result is shown in Fig. 5A, as the concentration of BaP increased from 0 to 1000 μg L^−1^ (0 to 3.96 μmol L^−1^), the fluorescence intensity decreased, resulting in about 80% loss of fluorescence signal at the highest concentration of BaP tested. The raw data was analyzed with the log–logit fitting method and the calculated Limit of Detection (LOD) (which made the signal equal to zero signal minus three times the standard deviation of the zero signal) from the competition curve was 0.32 ng mL^−1^ (Fig. 5B). The working range of the RFIA is 0.46 to 333 ng mL^−1^, which is much wider than conventional ELISA. To demonstrate the superiority of the new labeling strategy, we also constructed the biotin antibody/streptavidin/biotin–DNA immunoassay system of BaP and obtained a calculated LOD of 0.73 ng mL^−1^. In comparison, the LOD of the covalently attached immune complex system is lower. Moreover, for other immunoassay methods that have been reported so far, the redox-labeled electrochemical immunoassay method has an LOD of 2.4 ng mL^−1^ and a competitive piezoelectric biosensor with 1 μmol L^−1^.^14,33,34^ In terms of LOD, the RFIA method is more sensitive than these methods.

Then the BaP in 3R4F mainstream smoke was analyzed with the RFIA method and it was also measured under the same conditions using the most common conventional GC-MS method for comparison. As a result, it was found that the amount determined by the RFIA method was 12.16 ± 1.34 ng per cig, while the amount determined by GC-MS method was 8.32 ± 0.65 ng per cig. The results of the two methods were similar, but the operation of the RFIA method can be performed by one-step reaction, making the analysis simpler than the previous methods. To further evaluate the accuracy of the RFIA method, three different concentrations of BaP were added to the extracted 3R4F DMSO solution and then diluted with PBS and analyzed using immunoassay. Table 2 shows the test results and the recovery, and the results show that the RFIA has good accuracy and small matrix effects.

**Table 1:** Smoking parameters applicable to the method

Smoking regime	Puff volume (mL)	Puff frequency (s)	Puff duration (s)
ISO 3308:2012	35	60	2

**Table 2:** Recovery of BaP in spiked 3R4F DMSO extracted samples measured by the proposed immunoassay

Compound	Spiked concentrations (μg L^−1^)	Immunoassay^a^ (μg L^−1^)	Recovery (%)
Benzo[*a*]pyrene	0	10.21	—
	10	22.34 ± 2.47	121.30
	50	56.17 ± 4.51	91.92
	100	105.65 ± 7.12	95.44

a Mean (SD); *n* = 3.

### Cross-reactivity of other PAHs

Mainstream cigarette smoke contains six other PAHs with different numbers of benzene rings, naphthalene, anthracene, pyrene, benzo[*a*]anthracene, chrysene, and benzo[*b*]fluoranthene. The six compounds were tested with the RFIA system and competition curves were created, which were then compared with that of benzo[*a*]pyrene to assess the selectivity of the RFIA system. IC_50_ values were shown in Table 3, the IC_50_ value of BaP was set to be 100%. The results showed that the immunoassay of 2-ring and 3-ring PAHs, naphthalene and anthracene, had a very small cross reaction (<1%). The four-ring compounds, pyrene, ben[*a*]anthracene and chrysene, had moderate cross-reactivity (15, 22 and 31% for pyrene, ben[*a*]anthracene and chrysene, respectively). However, the benzo[*b*]fluoranthene, which possessed five rings like BaP, had a very high cross-reactivity of 61%. This may be the reason why the value of BaP in smoke measured by the RFIA method is higher than that of GC/MC assay. The main cause of cross-reactions is due to the non-specific recognition of BaP by antibodies. In our future work, more excellent antibodies will be try to prepared to increase the specificity of antibody recognition and thus achieve lower cross-reactivity in the RFIA. In the current situation, although cross-reactions have occurred in the RFIA, the test results are acceptable and consistent with previous reports.

**Table 3:** Cross-reactivities (CRs) of BaP antibody with 7 polycyclic aromatic hydrocarbons in mainstream cigarette smoke

Compound	Structure	CR (%)
Benzo[*a*]pyrene	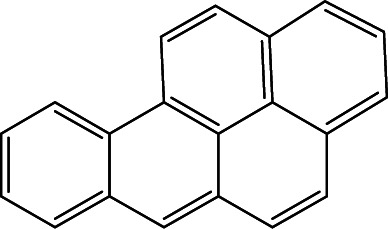	100
Naphthalene	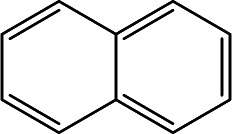	<1
Anthracene	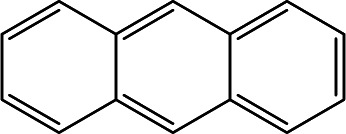	<1
Pyrene	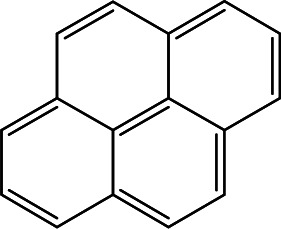	15
Ben[*a*]anthracene	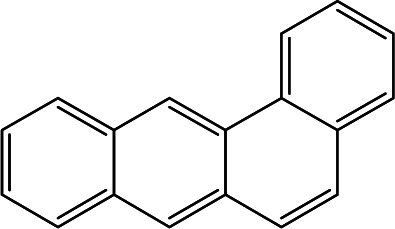	22
Chrysene	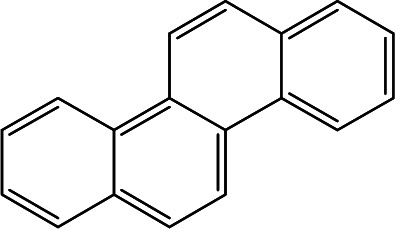	31
Benzo[*b*]fluoranthene	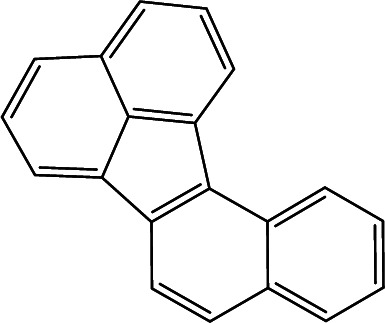	63

## Materials and methods

### Reagents and materials

Acenaphthene, benzo[*a*]pyrene, benzo[*a*]anthracene, benzo[*b*]fluoranthene, benzo[*j*]fluoranthene, chrysene, *n*-succinimidyl-*S*-actyl-thioacetate (SATA), sulfosuccinimidyl-4-(*N*-maleimidomethyl)cyclohexan-1-carboxylate (sulfo-SMCC), fluorescein isothiocyanate isomer I, and bovine serum albumin (BSA) were all purchased from Sigma-Aldrich (St. Louis, MO, USA). 1-[3-(Dimethylamino)propyl]-3-ethylcarbodiimide (EDC) and *N*-hydroxysuccinimide (NHS) were purchased from J&K chemical (Beijing, China). *Anti*-benzo[*a*]pyrene monoclonal antibody was purchased from Santa Cruz biotechnology (California, DA, USA). Taq DNA polymerase, DL 2000 DNA ladder, and DNA fragment purification kit were obtained from Takara Biotech Co. (Dalian, China). The primers were obtained from Sangon Biotech. Co., Ltd (Shanghai, China). ExpressPlus™ PAGE gels was from Genscript (Nanjing China). Slide-A-Lyzer™ mini dialysis device, SYBR Green I, and controlled protein–protein crosslinking kit were provided by Thermo Scientific (Rockfold, IL, USA). 3R4F reference cigarettes were purchased from University of Kentucky. All buffers were prepared using ultrapure water produced by a Milli-Q system (Millipore, Bedford, MA, USA).

### Preparation of PAH–BSA hapten

1-Pyrenebutyric acid was coupled with BSA and used as antigen. Briefly, 1-pyrenebutyric acid, NHS, and EDC were mixed together in 0.2 mL DMF, with a final concentration of 50 μmol L^−1^, 50 μmol L^−1^, and 60 μmol L^−1^, respectively. And the mixture was incubated at room temperature for 4 h to obtain the activated ester. A BSA solution of 5 mg mL^−1^ was prepared in sodium carbonate-bicarbonate buffer (50 mmol L^−1^, pH 9.6). Then 40 μL of the above activated ester was added very slowly in 5 μL aliquots while gently and continuously stirring the BSA solution, then the mixture was incubated at 4 °C for 8 h. Then, NH4Cl was added to a final concentration of 50 mmol L^−1^ to terminate the reaction, and the mixture was further incubated at room temperature for 2 h. After reaction completion, the mixture was then transferred into a desalting column to separate the PAH–BSA hapten.

### Preparation of –NH_2_ modified long chain DNA

The long chain DNA, a 251 bp fragment of *Phytophthora parasitica* var. *nicotianae*, was synthesized using the PCR protocol, the primers were designed using the Primer 5.0, and the sequences were as follows: 5′-NH_2_–C_12_-TGAACGCATATTGCACTTCC-3′(sense) and 5′-GACAAACCAGTCG CCAATTT-3′(antisense). PCR amplification was performed in such a condition: denature at 94 °C for 3 min, and then 35 cycles of denature at 94 °C for 30 s, annealing at 55 °C for 30 s and extension at 72 °C for 1 min, lastly extension at 72 °C for 5 min. The –NH_2_ modified long chain DNA was purified by phenol–chloroform extraction, and the yield was measured at 260 nm using the Thermo Nanodrop, and the concentration was adjusted to 7 μmol L^−1^ (about 1200 ng μL^−1^) for further use. The obtained –NH_2_ modified long chain DNA was characterized by 1.0% agarose gel electrophoresis.

### Conjugation of monoclonal antibody to –NH_2_ modified long chain DNA

For covalent conjugation of *anti*-benzo[*a*]pyrene monoclonal antibody to –NH_2_ modified long chain DNA, a coupling protocol described by Fischer *et al.* was used with minor modification.^35^ Briefly, the BaP monoclonal antibody (5 mg mL^−1^, about 33.5 μmol L^−1^) was incubated with a ∼10 fold molar excess of SATA, introducing the latent sulfhydryl groups to primary amines onto the antibody's lysine residues. The –NH_2_ modified long chain DNA was activated with a ∼10 fold molar excess of sulfo-SMCC. The activated DNA and antibody were both purified using the Slide-A-Lyzer™ mini dialysis device, and the concentration of DNA and antibody were measured at 260 nm and 280 nm, respectively. DNA and antibody were adjusted to equal molar concentration, and the conjugate reaction was started by adding the hydroxylamine–HCl into modified antibody, followed by mixing with activated NH_2_–DNA, and the mixture was incubated at room temperature for 1 h, the products were purified by polyacrylamide 6000 desalting columns. After denatured at 90 °C for 10 min, the antibody–DNA conjugate was characterized by 4–20% polyacrylamide gel electrophoresis.

### Immunoassay fabrication for BaP detection

The competitive immunoassay utilized a 96-well plate format. Firstly, PAH–BSA solution was diluted by sodium carbonate-bicarbonate buffer (50 mmol L^−1^, pH 9.6) to a concentration of 10 μg mL^−1^, 100 μL of diluted PAH–BSA was added to each well and incubated overnight at 4 °C. After being washed three times with 20 mmol L^−1^ pH 7.4 PBS buffer containing 0.1% (v/v) Tween 20, the plate was blocked with 20 mmol L^−1^ pH 7.4 PBS buffer containing 1 wt% BSA overnight at 4 °C. Then the plate was washed three times, and 50 μL antibody–DNA conjugate and 50 μL BaP standards with different concentrations were added into each well, and incubated at 37 °C for 1 h under shaking. Finally, SYBR Green I was added and incubated at room temperature for 5 min. After wash to remove unbound dyes, fluorescence intensity was measured on a Horiba FM-4 fluorescence spectrometer (Edison NJ USA) with 490 nm excitation, 525 nm emission.

### Characterization of the antibody–DNA conjugate by SDS-PAGE

ExpressPlus™ PAGE gels was purchased from Genscript (Nanjing, China). 5× loading buffer: 10% w/v SDS, 20% v/v glycerol, 0.05% w/v bromophenol blue, 10 mmol L^−1^ mercapto-ethanol, 0.2 mol L^−1^ Tris–HCl, pH 6.8; running buffer: 25 mmol L^−1^ Tris–HCl, 200 mmol L^−1^ glycine, 0.1% (w/v) SDS. Sample preparation: 2 μL antibody–DNA conjugate was added to 2 μL 5× loading buffer, deionized water was used to adjust the final volume to 10 μL. Next, the sample mixture was heated to 90 °C for 10 min. Then the sample was centrifuged briefly before loading the samples, and then electrophoresis was performed using purchased 5% polyacrylamide gel.

### Detection of BaP in mainstream cigarette smoke, recovery and cross reactivity study

The fabricated competitive immunoassay was further employed for screening of BaP in the total particulate matter (TPM) extract of mainstream cigarette smoke. First, twenty 3R4F reference cigarettes were smoked on a rotary smoking machine according to the standard smoking regimen described in ISO 3308:2008 (see Table 1). TPM was trapped with a 92 mm Cambridge filter pad and was extracted with 25 mL DMSO under mechanical shaking for 1 h. The extract was diluted by 10 times with PBS buffer (20 mmol L^−1^, pH 7.4) and then analyzed by the immunoassay. Meanwhile, TPM was extracted with 100 mL cyclohexane under mechanical shaking for 1.5 h, and then purified on a solid-phase extraction cartridge and further eluted with two 15 mL aliquots of cyclohexane. The eluent was evaporated to dryness and redissolved in 1 mL cyclohexane for GC/MS analysis.

To further evaluate the accuracy of the immunoassay, recovery study was conducted by spiking three different concentration levels of BaP into the TPM extract of 3R4F, after dilution with PBS, the extract was finally analyzed using the proposed immunoassay.

In addition, to assess the specificity, six other PAHs present in mainstream cigarette smoke, including naphthalene, anthracene, pyrene, benzo[*a*]anthracene, chrysene, and benzo[*b*]fluoranthene, were tested for cross-reactivity (CR) study. IC_50_ of BaP and these six PAHs were calculated from the competition curves, by setting IC_50_ value of BaP as 100% response, CRs of six other PAHs were evaluated.

## Conclusions

In this paper, a bifunctional complex with novel covalent antibody–DNA as analyte recognition and signal amplification was synthesized and used for competitive immunoassay of BaP. This RFIA method has good detection sensitivity and acceptable specificity when applied to the screening of BaP in cigarette mainstream smoke. The RFIA method enables identification and signal amplification in one step, simplifies the analysis process, and can provide potential applications in biochips and lateral flow platforms.

## Conflicts of interest

There are no conflicts to declare.

## Supplementary Material
